# Serotype and genetic diversity of human rhinovirus strains that circulated in Kenya in 2008

**DOI:** 10.1111/irv.12373

**Published:** 2016-03-02

**Authors:** Sylvia Milanoi, Juliette R. Ongus, George Gachara, Rodney Coldren, Wallace Bulimo

**Affiliations:** ^1^ College of Health Sciences (COHES)Jomo Kenyatta University of Agriculture and Technology (JKUAT)NairobiKenya; ^2^ Department of Medical Laboratory SciencesKenyatta UniversityNairobiKenya; ^3^ Department of Emerging Infectious Diseases (DEID)United States Medical Research Directorate‐ KenyaNairobiKenya; ^4^ A Special Field Activity of the Walter Reed Army Institute of ResearchSilver SpringMDUSA; ^5^ Department of BiochemistryUniversity of NairobiNairobiKenya

**Keywords:** Diversity, genetic, Kenya, rhinovirus, serotype

## Abstract

**Background:**

Human *rhinoviruses* (HRVs) are a well‐established cause of the common cold and recent studies indicated that they may be associated with severe acute respiratory illnesses (SARIs) like pneumonia, asthma, and bronchiolitis. Despite global studies on the genetic diversity of the virus, the serotype diversity of these viruses across diverse geographic regions in Kenya has not been characterized.

**Objectives:**

This study sought to characterize the serotype diversity of HRV strains that circulated in Kenya in 2008.

**Methods:**

A total of 517 archived nasopharyngeal samples collected in a previous respiratory virus surveillance program across Kenya in 2008 were selected. Participants enrolled were outpatients who presented with influenza‐like (ILI) symptoms. Real‐time RT‐PCR was employed for preliminary HRV detection. HRV‐positive samples were amplified using RT‐PCR and thereafter the nucleotide sequences of the amplicons were determined followed by phylogenetic analysis.

**Results:**

Twenty‐five percent of the samples tested positive for HRV. Phylogenetic analysis revealed that the Kenyan HRVs clustered into three main species comprising HRV‐A (54%), HRV‐B (12%), and HRV‐C (35%). Overall, 20 different serotypes were identified. Intrastrain sequence homology among the Kenyan strains ranged from 58% to 100% at the nucleotide level and 55% to 100% at the amino acid level.

**Conclusion:**

These results show that a wide range of HRV serotypes with different levels of nucleotide variation were present in Kenya. Furthermore, our data show that HRVs contributed substantially to influenza‐like illness in Kenya in 2008.

## Introduction

Human *rhinoviruses* (HRV) form one of the largest genera within the *Picornaviridae* family. They are non‐enveloped viruses with a linear positive sense, single‐stranded RNA genome of about 7200 bp. The viral genome is translated into a single polyprotein which is proteolytically cleaved to produce 11 proteins. These include four structural proteins (VP1, VP2, VP3, and VP4) which are used as target regions in the detection, species diversity, and serotype identification of HRV variants in diagnostic respiratory samples.

Since their discovery in the 1950s, over 100 serotypes have been confirmed.^1^ Initially, these were classified into two species; species A and B (HRV‐A and HRV‐B). From 2006, previously undetected strains were discovered in multiple studies around the world,^2^, ^3^, ^4^, ^5^ these have now been designated HRV‐C. HRV‐Cs have distinct characteristics that differentiate them from species A and B, but their specific pathogenic mechanisms have not yet been clearly defined.

Traditionally, HRVs are associated with upper respiratory infections also known as the common cold, which is mostly a self‐limiting illness. However, in recent years, with increased implementation of molecular assays in the detection of HRVs, they have been identified as etiological agents of lower respiratory infections and are closely associated with more serious clinical presentations including asthma, chronic obstructive pulmonary disease, fatal pneumonia, and bronchiolitis.^6^, ^7^, ^8^, ^9^, ^10^ The serious illnesses associated with HRV are mostly reported among children, immunocompromised adults, and the elderly. HRVs are present worldwide, all year‐round, and therefore account for a significant amount of viral respiratory tract infections. These result in restricted activities, work, and school absenteeism which, in turn, directly and indirectly lead to considerable economic burden.^11^, ^12^

Despite the economic and medical importance of HRV, little is known about their circulation dynamics and serotype diversity in Kenya. This study retrospectively employed a molecular approach to type and characterize HRVs present in Kenya in 2008.

## Materials and methods

### Study design and population

Samples were randomly selected using the systematic sampling technique 13 from archived samples collected as part of the respiratory virus surveillance program of the United States Army Medical Research Directorate‐Kenya (USAMRD‐K) at the National Influenza Center within the Kenya Medical Research Institute (KEMRI). These samples had been collected in 2008 from patients who had enrolled in the surveillance program for respiratory viruses. This program's network was designed to include different population demographics and geographic regions across Kenya and comprised: Malindi, New Nyanza, Isiolo, Alupe, Port Reitz, Kisii, Kericho, and Mbagathi hospitals.

Participants enrolled in the surveillance program were outpatients who presented with ILI symptoms and were >2 months old. Patient clinical data had been collected along with their demographic information. The ILI case definition included sore throat, cough, and temperature >38^°^C. Nasopharyngeal swabs were collected from each participant using a sterile flexible flocked swab (COPAN Diagnostics Inc., Murrieta, CA, USA) that was immediately inserted in a cryovial tube containing 1 ml of viral transport medium (VTM) and transported to the National Influenza Center Laboratory observing the cold chain.

All participants were appropriately informed of the study objectives by the attending study personnel and a written consent was obtained. This study was reviewed and approved by the Walter Reed Army Institute of Research (WRAIR) Institutional Review Board and the Kenya Medical Research Institute (KEMRI) Ethics Review Committee under protocol approvals WRAIR #1267 subproject 2 and KEMRI SSC #2188, respectively.

### RNA extraction, PCR amplification, and nucleotide sequencing

Viral RNA was extracted from 100 μl of each sample using QIAmp Viral RNA mini kit (Qiagen Inc., Valencia, CA, USA) according to the manufacturer's specifications. Preliminary detection of HRV was performed using real‐time RT‐PCR with primers and probes directed to the 5′ non‐coding region as previously described.^14^ HRV‐positive samples were amplified using RT‐PCR with primers flanking the hypervariable part of the 5′ NCR, the entire VP4 gene, and the 5′ terminus of the VP2 gene.^1^ The amplicons were purified using exonuclease 1/shrimp alkaline phosphatase (ExoSap‐II) enzyme (Affymetrix, Santa Clara, CA, USA). Direct amplicon sequencing was performed using the Big Dye chain terminator (v.3·1) (Applied Biosystems, Foster City, CA, USA) on an automated ABI 3500XL genetic analyzer (Applied Biosystems).

Sequences of the partial VP4/VP2 gene region of the Kenyan HRV strains described in this study were submitted to GenBank under the accession numbers: KR059989–KR060014.

### Sequence analysis

Consensus contig assembly of the newly determined nucleotide sequences was performed using dna baser sequence assembler V.4 (2013) (Heracle BioSoft, www.DnaBaser.com) and thereafter edited using BioEdit sequence alignment editor.^15^ Multiple sequence alignment was performed using muscle software V.3·8.^16^ This was achieved by comparing the analyzed sequence fragment to all the available *Rhinovirus* reference prototypes retrieved from Genbank (http://www.ncbi.nlm.nih.gov). Phylogeny inference was made using mr. bayes software V.3·2 17 and the generated tree was viewed using figtree V.1·4·0 software (http://tree.bio.ed.ac.uk/software/figtree/). Serotype identity was determined on the basis of a phylogenetic tree. Natural selection pressure analyses of the VP4/VP2 genomic region were performed on the online Data Monkey Interface (http://www.datamonkey.org).^18^ Selection pressure at individual codon sites and the mean d_N_/d_S_ rates of evolution were estimated using the single likelihood ancestor counting (SLAC), the fixed effects likelihood (FEL), and the internal fixed effects likelihood (IFEL) methods. The rate of synonymous nucleotide per non‐synonymous site (dN) against that of nucleotide substitution per synonymous site (dS), which is the dN/dS ratio, was used as an index to assess selection pressure and was interpreted as follows: dN/dS >1 = positive (diversifying) selection, whereas dN/dS <1 = negative selection (purifying) and dN/dS = 1 means neutral selection. The level of significance for selection was based on the *P*‐value. Strong evidence of selection was indicated by a *P*‐value < 0·05.

### Detection of other respiratory viruses

To detect other respiratory viruses, we used CDC protocols for molecular (PCR) detection of human influenzaviruses, human adenoviruses, human parainfluenzaviruses, human metapneumoviruses, hRSV, HSV1, coronaviruses (including SARS‐CoV and MERS‐CoV), and respiratory enteroviruses including coxsackieviruses.

## Results

A total of 517 samples collected in 2008 were randomly selected and retrospectively screened for HRVs. These were from patients aged between 3 months and 47 years old with a 9:4 male to female ratio. Clinical symptoms exhibited by these patients included the following: fever (100%), cough (100%), runny nose (92%), nasal stuffiness (69%), sputum production (38%), difficulty in breathing (35%), diarrhea (27%), vomiting (23%), and chills (19%) among others (Table 1). These samples were collected all year‐round except for January and December. However, March and May had the highest recordings with 39% of the positive samples having been collected in these months.

**Table 1:** Clinical Symptoms and demographic information of 26 patients positive for HRV

Sample ID	Gender	Age	Month of sample collection	Hospital	Symptoms
KENHRV001	F	1 y, 10 m	April	Malindi	Cough, fever, runny nose
KENHRV002	M	3 y, 3 m	February	Kisii	Cough, fever, chills, sore throat, runny nose
KENHRV003	M	2 y, 3 m	September	Kericho	Cough, fever, retro‐orbital pain, malaise, nasal stuffiness, runny nose
KENHRV004	F	8 m	March	Malindi	Cough, fever, diarrhea, difficulty breathing, vomiting, nasal stuffiness, runny nose
KENHRV005	M	4 m	February	Isiolo	Cough, fever, neurological, runny nose
KENHRV006	M	1 y	August	Isiolo	Cough, fever, nasal stuffiness, runny nose
KENHRV007	F	10 m	October	Port Reitz	Cough, fever, runny nose
KENHRV008	M	3 y, 5 m	February	Port Reitz	Cough, fever, abdominal pain
KENHRV009	M	2 y, 10 m	April	Port Reitz	Cough, fever, runny nose
KENHRV010	M	47 y, 11 m	March	Alupe	Cough, fever, sputum production, headache, joint pain, nasal stuffiness, runny nose
KENHRV011	F	1 y, 1 m	November	Malindi	Cough, fever, difficulty breathing, nasal stuffiness, runny nose
KENHRV012	M	10 m	March	Mbagathi	Cough, fever, nasal stuffiness, runny nose
KENHRV013	M	1 y, 10 m	May	Mbagathi	Cough, fever, sputum production, difficulty breathing, chills, abdominal pain, nasal stuffiness, runny nose
KENHRV014	M	2 y	May	Mbagathi	Cough, fever, sputum production, nasal stuffiness, runny nose
KENHRV015	M	2 y, 9 m	June	Mbagathi	Cough, fever, sputum production, vomiting, neurological, abdominal pain, nasal stuffiness, runny nose
KENHRV016	M	3 m	July	Mbagathi	Cough, fever, difficulty breathing, chills, vomiting, neurological, nasal stuffiness, runny nose
KENHRV017	F	3 y, 1 m	July	Mbagathi	Cough, fever, sputum production, diarrhea
KENHRV018	M	2 y	May	New Nyanza	Cough, fever, diarrhea
KENHRV019	F	3 m	September	Mbagathi	Cough, fever, sputum production, diarrhea, difficulty breathing, neurological, nasal stuffiness, runny nose
KENHRV020	M	8 m	November	Mbagathi	Cough, fever, sputum production, diarrhea, difficulty breathing, chills, vomiting, nasal stuffiness, runny nose,
KENHRV021	M	1 y, 10 m	May	New Nyanza	Cough, fever, diarrhea, vomiting, runny nose
KENHRV022	M	7 y	September	Port Reitz	Cough, fever, nasal stuffiness, runny nose
KENHRV023	M	4 m	March	Mbagathi	Cough, fever, difficulty breathing, vomiting, neurological, nasal stuffiness, runny nose
KENHRV024	F	3 y, 6 m	March	Mbagathi	Cough, fever, sputum production, difficulty breathing, nasal stuffiness, runny nose
KENHRV025	M	1 y, 11 m	April	Alupe	Cough, fever, nasal stuffiness, runny nose
KENHRV026	F	1 y, 2 m	May	Alupe	Cough, fever, difficulty breathing, nasal stuffiness, runny nose

y, Years; m, Months; M, Male; F, Female.

Among the samples, 131 (25%) tested positive for HRV by real‐time PCR. A fragment of ~549 bp including the hypervariable part of the 5′ NCR and full‐length VP4 and partial VP2 gene region was successfully amplified in 37 (28%) samples. Eleven samples were excluded from further analyses. Of these, six displayed poor sequence quality while the other five sequences comprised echovirus (two) and coxsackieviruses (three). BLAST analyses of the remaining 26 sequences indicated the three HRV species comprising of 14 HRV‐A, 9 HRV‐C, and 3 HRV‐B species.

All the patient samples had been previously tested for other viral etiologies of respiratory disease. From those tests, the two patients infected with KENHRV002 and KENHRV022 had coinfection with Coxsackie B virus and one patient infected with KENHRV026 had a coinfection with human parainfluenzavirus type 3.

**Figure 1:**
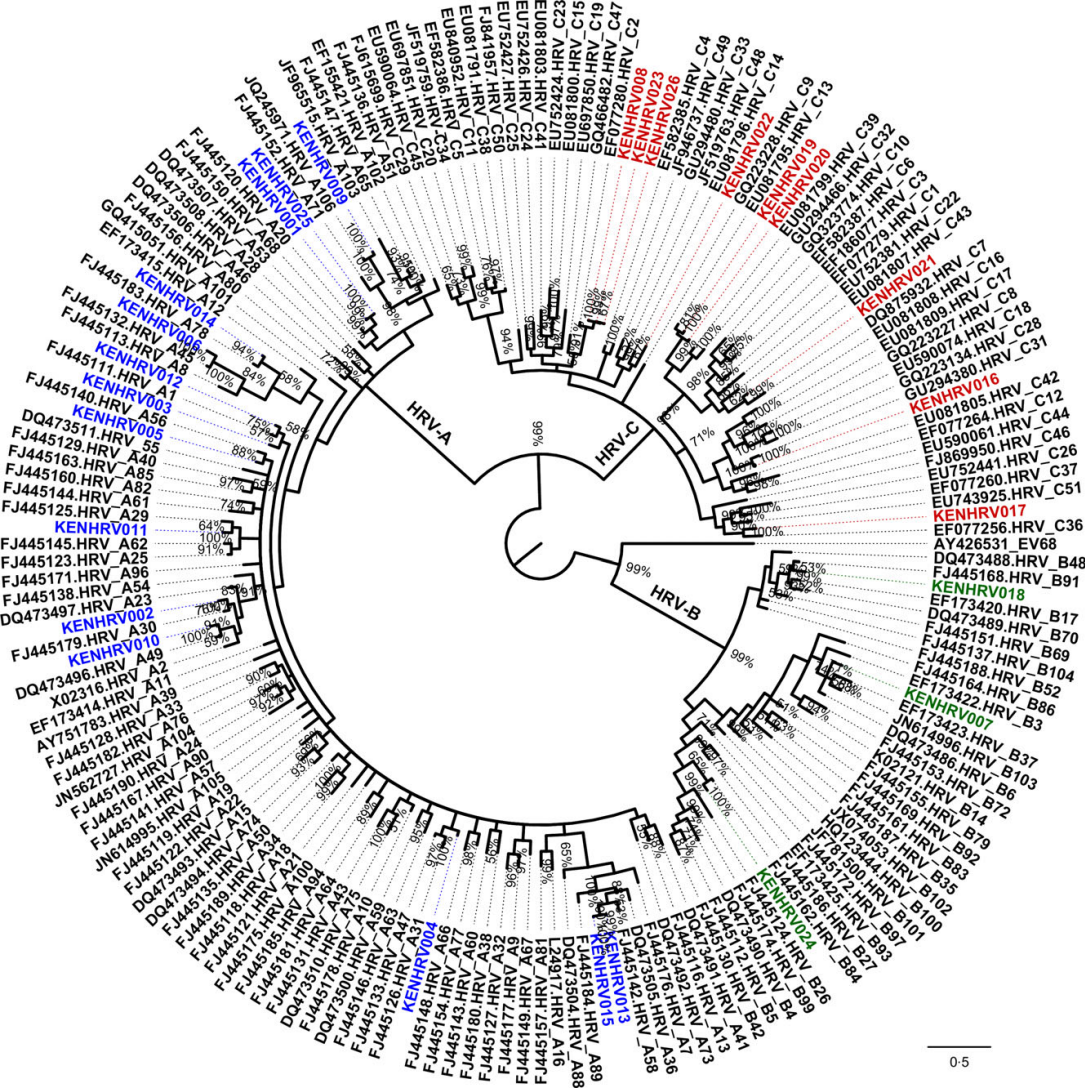
Phylogenetic tree based on nucleotide variations in the VP4/VP2 gene region of Human rhinovirus strains. The Kenyan strains are designated with the prefix ‘KEN’, whereas the reference strains are represented by their GenBank accession numbers. The three species are represented in color: blue, HRV‐A; red, HRV‐C; green, HRV‐B.

Analyses of genetic identities revealed that intraspecies identities of the Kenyan HRV strains ranged from 58% to 100% at the nucleotide level and 55% to 100% at the amino acid level. Within the HRV‐A species, nucleotide identity of the Kenyan viruses to their prototype reference strain ranged from 77% to 88% and the corresponding amino acid identities were between 91% and 99%. These strains also shared 73–96% nucleotide and 87–99% amino acid identities with other HRV‐A contemporaneous strains. HRV‐B strains displayed 77–97% and 87–97% sequence similarity to their prototype reference strain at the nucleotide and amino acid levels, respectively, and 75–88% nucleotide identities and 85–99% amino acid identities with other HRV‐B contemporaneous strains. There was a slight difference in the sequence comparison of the HRV‐C strains to the reference and the prototype strains. These showed a much lower relatedness compared to the other two species. Percentage nucleotide identity to the prototype reference strain was only 73–77% and 82–92% at the amino acid level. Furthermore, comparison of the Kenyan HRV‐C strains to contemporaneous strains showed the nucleotide and amino acid identities varying between 59–98% and 60–100%, respectively. Phylogenetically, the Kenyan HRVs clustered into 20 serotypes among the HRV‐A, HRV‐B, and HRV‐C species (Figure 1). Serotype‐specific comparison of the Kenyan HRV sequences to contemporaneous sequences and the serotype‐specific reference sequence available in Genbank revealed that the nucleotide and amino acid identities generally ranged between 79–100% and 94–100%, respectively (Table 2).

**Table 2:** Serotype‐specific nucleotide and amino acid identities comparison of Kenyan HRVs to contemporaneous and reference sequences

Species and serotype	% Nucleotide identity	% Amino acid Identity
A01	88–100	100
A20	90–96	97–99
A29	88–90	100
A30	90–98	98–100
A45	92	98
A47	90–97	99–100
A49	92–98	98–100
A56	91–98	98–99
A58	90–98	98–100
A71	81–97	96–99
A78	89–98	100
B06	84–93	99–100
B84	93	99–100
B91	87–88	97–98
C02	92–94	99–100
C07	86–87	96
C13	92–94	99–100
C31	93	99
C33	79	82
C36	83–86	94

The Kenyan HRV strains showed countrywide distribution with more than one serotype circulating in the same region. The coastal region had the highest number of serotypes in circulation with seven identified (A01, A20, A47, A49, A71, C02, and C33), followed by the Nairobi region where six (A58, B84, C02, C13) serotypes circulated. Five different HRV serotypes (A30, A20, A29, C02, and B91) were identified from the western region at the Kisii, Alupe, and New Nyanza hospitals. Only one serotype (A01) was identified from the Rift Valley region.

Selection pressure analyses of the VP4/VP2 genomic region revealed no statistically significant (*P*‐value < 0·05) positive selection as determined by the SLAC, FEL, or IFEL methods. However, numerous negatively selected sites were observed for all the species. The global selection pressures on the gene region for HRV‐A, HRV‐B, and HRV‐C as estimated by the SLAC method were 0·0353, 0·0305, and 0·0702, respectively (*P*‐value < 0·05).

## Discussion

HRVs are etiological agents of lower respiratory infections closely associated with serious clinical presentations including asthma, chronic obstructive pulmonary disease, fatal pneumonia, and bronchiolitis,^6^, ^7^, ^8^, ^9^, ^10^ yet there is a dearth of information regarding their circulation dynamics and serotype diversity across different regions in Kenya. In this study, we sought to address this gap and found that in 2008, all the three species of HRVs were detected in patients who presented with ILI symptoms in hospitals across Kenya. Whereas symptoms data for all patients were available, no clinical diagnosis data were provided by the attending clinicians and therefore it was not possible to associate HRV infection with disease severity. However, detection of HRV in symptomatic patients enrolled in this study suggests that in Kenya, HRVs contribute to diseases that manifest as ILI. Indeed, some studies have reported more frequent HRV detection in symptomatic than asymptomatic cases.^19^, ^20^

In 2008, HRV‐A was the predominant strain circulating in Kenya followed by HRV‐C and the least common was HRV‐B. Studies in other countries reported similar predominance of HRV‐A between 2009 and 2012 in the United States, Japan, UK, and the Netherlands,^21^, ^22^, ^23^, ^24^ yet others have reported HRV‐B as the predominant species in Spain and Thailand in the same period.^6^, ^25^ Recently, a higher prevalence of HRV‐C over HRV‐A was observed in Australia among preschool‐aged children.^26^ Whereas the majority (96%) of the patients in this study were children, it is known that the distribution patterns of HRV may vary depending on population under study.^27^, ^28^

Phylogenetic analysis confirmed circulation of 20 diverse HRV serotypes in Kenya in 2008. The most predominant serotype was C02 (three) followed by C13, A01, and A20 all of which had two strains detected, while all the other serotypes recorded one strain each. This is in contrast to findings in Mongolia where the most frequently detected HRV serotypes were A12, A46, A78, A80, B6, B35, B52, B86, C2, C5, and C4 between the years 2008 and 2013.^29^ Furthermore, our findings here were limited to a period of 1 year and may not represent a good comparator.

Despite their recent discovery, HRV‐Cs are not newly emerged because they have been in circulation as far back as 1982.^30^ Infections with the novel HRV‐C are associated with severe disease outcomes compared to those with HRV‐A and HRV‐B.^6^, ^7^, ^8^, ^9^, ^10^ In this study, six cases of HRV‐C were detected within the year. 83% of these cases were acute, presenting with difficulty in breathing confirming severity of diseases associated with these species.

Natural selection pressure analysis of the VP4/VP2 gene region from all the Kenyan HRV strains identified many negatively selected sites. This indicates that they have evolved through negative purifying selection which works by removing deleterious mutations. The numerous negatively selected sites found in the Kenyan HRV strains may indicate that portion of the genome has attained genetic stability and therefore resist deleterious mutations. These findings echo those of previous reports.^31^, ^32^

This study had three major limitations. First, due to the retrospective approach of the study, it was not possible to include a control group of asymptomatic patients from the same period. The control group would have facilitated association of a particular HRV type with severity of disease. Secondly, the sampling period was only limited to 1 year which could have lead to underestimation of the type diversity overall. A longer study observation period may have revealed a different picture. Finally, the lack of clinical diagnosis data for patients enrolled in this study hampered efforts to link HRV infection to disease severity. Notwithstanding these limitations, this is the first study cataloging a year‐long countrywide circulation of HRV species and serotypes in Kenya.

In conclusion, we have for the first time confirmed that HRVs are associated with ILI across Kenya and demonstrated the broad range of HRV serotypes in circulation. Furthermore, we have also demonstrated marked nucleotide variation in the VP4/VP2 junction among the Kenyan viruses. In future, comprehensive characterization of HRVs circulating in Kenya should involve full genome sequencing of the viruses.
